# USING INTERPERSONAL RELATIONSHIPS AND SOCIAL NETWORK TO UNDERSTAND ELDER MISTREATMENT RECURRENCE

**DOI:** 10.1093/geroni/igad104.2908

**Published:** 2023-12-21

**Authors:** Wei- Lin Xue, Zachary Hass, Pi-Ju Liu

**Affiliations:** Purdue University, West Lafayette, Indiana, United States; Purdue University, West Lafayette, Indiana, United States; Purdue University, West Lafayette, Indiana, United States

## Abstract

The incidence of elder mistreatment are often recurrent rather than one-time. The recurrence of mistreatment contributes to severe physical and psychological problems for victims and negative social consequences. Poor interpersonal relationships and social isolation are the main factors of mistreatment occurrence in the literature. However, very few studies have considered how the quality of interpersonal relationships and the level of social networks affect the likelihood of mistreatment recurrence. We used the National Social Life, Health, and Aging Project waves 1 (2005–06) and 3 (2015–16) data for longitudinal analyses (N=1,592). The quality of interpersonal relationships (i.e., supportive relationship, strained relationship) and the level of social networks (i.e., social engagement, social isolation, and feeling safe in society) were conceptualized using factor analysis to estimate factor scores. Logistic regression models were built to predict the risk of recurrent mistreatment using interpersonal relationship quality and the level of the social networks. Controlling for demographic variables, victims in wave 1 with support from interpersonal relationships are more likely to experience reoccurring mistreatment (p< 0.05, OR=1.58). Model interaction effects indicated that older adults with strained interpersonal relationships and low social engagement are most at risk of experiencing mistreatment recurrence. The surprising finding that supportive interpersonal relationships are positively associated with mistreatment recurrence might indicate that those who support older adults are potential perpetrators. Identifying potential perpetrators and assessing the quality of interpersonal relationships and social networks are essential to preventing mistreatment recurrence.

